# Electrospun Nanomaterials Based on Cellulose and Its Derivatives for Cell Cultures: Recent Developments and Challenges

**DOI:** 10.3390/polym15051174

**Published:** 2023-02-26

**Authors:** Kristina Peranidze, Tatiana V. Safronova, Nataliya R. Kildeeva

**Affiliations:** 1 Department of Materials Science, Lomonosov Moscow State University, Leninskie Gory 1, Building 73, 119991 Moscow, Russia; 2 Department of Chemistry, Lomonosov Moscow State University, Leninskie Gory 1, Building 3, 119991 Moscow, Russia; 3 Department of Chemistry and Technology of Polymer Materials and Nanocomposites, The Kosygin State University of Russia, Malaya Kaluzhskaya 1, 119071 Moscow, Russia

**Keywords:** scaffolds, electrospun nanofibers, cellulose, extracellular matrix, cell culture

## Abstract

The development of electrospun nanofibers based on cellulose and its derivatives is an inalienable task of modern materials science branches related to biomedical engineering. The considerable compatibility with multiple cell lines and capability to form unaligned nanofibrous frameworks help reproduce the properties of natural extracellular matrix and ensure scaffold applications as cell carriers promoting substantial cell adhesion, growth, and proliferation. In this paper, we are focusing on the structural features of cellulose itself and electrospun cellulosic fibers, including fiber diameter, spacing, and alignment responsible for facilitated cell capture. The study emphasizes the role of the most frequently discussed cellulose derivatives (cellulose acetate, carboxymethylcellulose, hydroxypropyl cellulose, etc.) and composites in scaffolding and cell culturing. The key issues of the electrospinning technique in scaffold design and insufficient micromechanics assessment are discussed. Based on recent studies aiming at the fabrication of artificial 2D and 3D nanofiber matrices, the current research provides the applicability assessment of the scaffolds toward osteoblasts (hFOB line), fibroblastic (NIH/3T3, HDF, HFF-1, L929 lines), endothelial (HUVEC line), and several other cell types. Furthermore, a critical aspect of cell adhesion through the adsorption of proteins on the surfaces is touched upon.

## 1. Introduction

Over the past two decades, various natural and synthetic polymers have gained a lot of attention as biocompatible biodegradable components for the production of materials with nano- and microscale fibrous architecture intended for use in a wide range of applications, including such rapidly evolving fields as drug delivery [1], wound healing [2], cellular agriculture [3,4] and tissue engineering [5]. In certain cases, related to cell culturing, the investigation of polymers is aimed at the fabrication of nanofibrous 3D matrices that imitate the features of extracellular matrix (ECM) present in human connective tissues (dermis, muscle, bone, cartilage tissues, etc.) [6]. Polysaccharides, long-chain bio-based molecules, performing storage (starch), and structural (chitin, cellulose) functions in living organisms show high potential in biomedical applications due to their beneficial properties (high versatility and compatibility with cells, including anti-inflammatory and anti-microbial effects) compared to numerous synthetic polymers. Among all kinds of polysaccharides, cellulose is considered one of the most significant biodegradable biopolymers widely distributed in nature [7].

Cellulose has an unbranched structure with the repeating (C_6_H_10_O_5_)_n_ units that build up macromolecules. It is commonly presented in the form of microfibrils in the cell walls of wood and plant, algae tissues, as a membrane, tunicate epidermal cells, or as a product of bacteria vital activity, and its derivatives can be easily produced by simple reactions between active hydroxyl groups and functional substituents [8]. Cellulose-based materials possess a semi-crystalline structure composed of superfine fibrils with repeating large ordered and small disordered domains with a percentage of crystalline and amorphous phases depending on the method of the material’s synthesis. The multi-level structure of cellulosic materials that spread from the nanoscale to the microscale ensures their appealing mechanical properties, including the specific strength of cellulose-based fibers [9]. Thus, along with biocompatibility, renewability, functionality, and environmental friendliness, the unique mechanical features of filaments make cellulose and its derivatives quite attractive for use in certain biomedical areas.

The tendency to replicate the natural ECM’s fibrous structure with appropriate filament thickness and alignment led to the development of several important techniques for scaffold fabrication that involve viscous solution systems of polymers under study. A biomimicking approach in tissue engineering is aimed at the development of a substrate made of biocompatible biodegradable constituents of a suitable phase composition with surface morphology and roughness that provide essential cell adhesion and proliferation. Some advanced biomedical applications involve the use of bioactive and/or antibacterial drugs incorporated in polymeric systems with controlled release properties, which requires the materials to be functionalized. Additionally, the mechanical features (overall stability, tensile stress, flexibility, etc.) in combination with permeable architecture play a substantial role when interacting with biological fluids. The most common techniques used in bioscaffolding include electrospinning [10,11,12], touch-spinning [13], spinneret-based tunable engineered parameters (STEP method) [14] techniques, as well as several conventional methods, such as 3D printing or simple extrusion from polymeric solutions [15]. The high demand for scaffolds to possess hierarchical structures at the nanoscale level makes conventional methods less favorable since the obtained fibers are large microscale objects incapable of efficient cell capture. Various electrospinning techniques have shown relatively good results in producing fibers with structural stability and large surface area per volume that enables sufficient cell attachment [16,17]. However, poor alignment of electrospun fibers impedes the fabrication of nanomaterials with controllable fiber thickness and mechanical strength in different directions. In addition, mechanical strength assessment for the single filament is considered a rather challenging task that can be resolved in a limited way using special devices for tensile strength measurement. Nevertheless, electrospinning as a facile and efficient technique to produce biodegradable mats consisting of stable randomly arranged fibrous networks attracts significant interest in biomedical areas and cellular agriculture.

The study of electrospun materials based on cellulose and its derivatives opens up a wide range of applications in tissue engineering and cellular agriculture, where the core goal is to obtain a material (scaffold) with suitable composition and structural features enabling high cell adhesion and continuous cell growth. The study of 3D structure influence on cell growth and migration into the volume of the scaffold appears to be another key task of bioscaffolding-related research. To date, cellulosic electrospun nanomaterials have been investigated as substrates for cell culturing in bone and cartilage [18], vascular [19], muscle [20], skin [21], etc. tissue engineering. Their potential for application in tissue engineering together with certain surface modifications, such as treatment with morphogenetic proteins (growth factors) [22] or fibronectin (adhesive protein) [23], lays the foundation for further material improvement for cell culturing. Another comparatively young branch of research associated with the fabrication of scaffolds is cellular agriculture. This area is mainly focusing on the large-scale production of artificial ECM analogs designed for the growth of alternative proteins actively used in the food industry, where over the past few years the increasing demand for synthetic meat and seafood products has gained considerable attention. Special interest in cellulosic materials is also justified by the cost-effectiveness and market prevalence [24].

Currently, the nanofiber scaffolds based on cellulose and its derivatives are subject to a number of requirements that determine their multipurpose use in medicine. One of the trends in tissue engineering is the design of scaffolds with the delivery potential for growth factors, cytokines, and cell adhesion peptides uniquely binding to cell receptors [25], as well as anti-inflammatory and antimicrobial agents. The bioscaffolds with such incorporations can be involved in innovative gene therapy tactics with the utilization of DNA encoding for therapeutic genes, which is a breakthrough in the sustained release of therapeutic factors and hence in the promoted tissue healing process [26]. Remarkably, another significant aspect in tissue engineering directions is the increased cell adhesion derived from surface charge tuning due to the presence of specific functional groups. A few examples of the surface charge effect on cell attachment will be given in *Section 5. Applicability of Electrospun Nanomaterials in Cell Culturing*. The behavior of various cell lines toward different cellulosic materials remains unexplored, and the study of the tendency to adhere to surfaces exhibiting different ζ-potential is a crucial task of interdisciplinary research. It is important to note that the cell adhesion on the outside of the material should not occur too rapidly in order to avoid the formation of a necrotic core that prevents the transport of nutrients from the media to the inner parts of the scaffold. In this context, the studies [26,27] emphasize vascularization as one of the significant criteria when designing cellulosic constructs for cardiovascular tissue engineering. Thus, the development of vascular networks will allow for angiogenic factor delivery. However, the regulation of the fiber diameter at the nanoscale level for this purpose is a complex materials science task, which at the moment cannot be solved by electrospinning techniques. 

To date, the widespread trends in tissue engineering approaches include the creation of three-dimensional cell culture systems that are supposed to stimulate physiological conditions to a greater extent compared with conventional 2D systems. Therefore, 3D nanofibrous constructs based on cellulose and its derivatives are of great interest in scaffold fabrication via electrospinning methods.

The current minireview is focusing on recent advances in bioscaffolding related to the fabrication of cellulose-based electrospun materials, their benefits and drawbacks compared to fibrous structures obtained by other methods, and applicability toward cell cultures. The key features of electrospinning techniques, including solvent and solution properties, as well as spinning conditions, are discussed. Therefore, the review provides general information about the prospects of the potential application of such materials in the field of bioscaffolding.

## 2. Cellulose and Its Derivatives as Multifunctional Materials

Cellulose is a polysaccharide composed of an unbranched chain of several hundred to over ten thousand D-glucose units linked together by β(1→4) bonds. Each glucose residue contains three hydroxyl groups participating in the intra-chain hydrogen bonding with the oxygen of the adjoining ring, which stabilizes the linear conformation of the polymeric chain [28]. Strong intramolecular hydrogen bonds and Van der Waals forces are responsible for the aggregation of cellulose chains with lateral dimensions up to several nm [29]. Simple reactions between hydroxyl groups and various functional substituents may result in a wide spectrum of cellulose derivatives. The properties of cellulose derivatives improved due to certain functional groups leading to a better quality of the solutions used for electrospinning [30]. A fairly accurate classification of cellulose ether and ester derivatives is given in [31]. Based on the study and several recent research articles, the most frequently discussed cellulose derivatives can be presented in Table 1.

A semi-crystalline nature of cellulosic materials results in a different percentage of highly ordered crystalline and unordered amorphous regions, which depends on the origin or treatment method of raw materials. Thus, higher degrees of crystallinity (80–100%) are observed in bacteria-produced cellulose, while plant-based cellulose shows a degree of crystallinity up to 60%. Four types of allomorphs of crystalline cellulose (I–IV) are described in [32].

**Figure 1:**
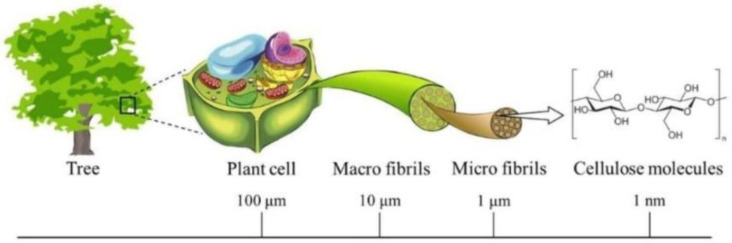
The hierarchical structure of plant-based cellulose at different scale levels [33].

The desire to replicate the fibrous architecture of a natural matrix requires a better understanding of the hierarchical organization of cellulosic materials. According to a study [33], cellulose hierarchical forms can be classified into cellulose fibers, cellulose filaments, cellulose crystals, and cellulose nanofibrils. The hierarchical structure of plant-based cellulose is reflected in Figure 1. Microfibrils of cellulose found in nature are usually assembled into macroscopic cellulose fibers with different geometry types and lengths ranging from several mm to hundreds of cm, which is a good model for the production of fibers in the textile industry. In the framework of this research, special attention will be paid to the cellulosic nanofibers obtained by chemical ways, in particular the electrospinning method. Typically, electrospun cellulose nanofibers are considered to have a diameter in the range of 5–100 nm (potentially much greater) and a fiber length reaching several tens of cm. However, the majority of research works apply the term “nano” to fibers with a diameter of 50–5000 nm [34]. Nanofibrous materials obtained by various electrospinning techniques are usually non-woven mats of fibers with comparatively high interconnectivity and special porosity different from that of materials obtained by leaching methods.

The scope of application of fibers based on cellulose and its derivatives, of course, cannot be limited to the development of scaffolds for cell culturing. Along with the widespread use of cellulosic fibers in the textile and paper industries, such materials find their application as sensors [35], electro-conductive materials [36], wound healing materials [37], as well as materials for water treatment [38] and active packaging [39]. Thus, in a study [40], the authors showed that ethyl hydroxyethylcellulose functionalized by 4-(2-(pyridine-4-yl)vinyl)phenol and 4-[4-(dimethylamino)styryl]pyridine could be used to prepare electrospun nanofibers for the detection of CN^−^ groups in aqueous solutions. In addition, electrospun cellulose acetate nanofibers with covalently bonded protoporphyrin IX were demonstrated to have great potential for ammonia sensing, which can be used to monitor the freshness of seafood [41]. Lyu et al. [42] reported moisture-induced electricity generators based on cellulose acetate nanofibers with tunable porosity achieved by changing the time of thermal post-treatment. Cellulose-based nanofibrous membranes prepared by deacetylation of cellulose acetate fibers demonstrated fascinating efficiency of up to 99.5% for water/oil separation in order to reduce the amount of petroleum hydrocarbons released to aqueous systems [43,44]. All the examples allow us to consider cellulosic nanofibers as multifunctional materials with a high surface area-to-volume ratio and pore interconnectivity.

## 3. Electrospinning Challenges

A tremendous number of electrospinning techniques have been developed and applied in biomimicking approaches of tissue engineering to fabricate ECM-like scaffolds that provide sufficient cell attachment, growth, and differentiation. Today, more and more scientific works are focusing on the development of 3D-structured electrospun scaffolds and the influence of the three-dimensional environment on cell behavior [45]. Such a complex structure can be potentially formed by merging separate electrospun mats into a single biomaterial. In [46], the authors assume that one of the favorable conditions for cell capture/attachment is related to the optimal distance between single fibers in the structure, which is equal to ~20–80 µm, considering the cell size in the range of ~15–20 µm. Effective surface-to-volume ratio and pore interconnectivity of electrospun nanofibers mentioned above can ensure suitable conditions for cell adhesion.

**Figure 2:**
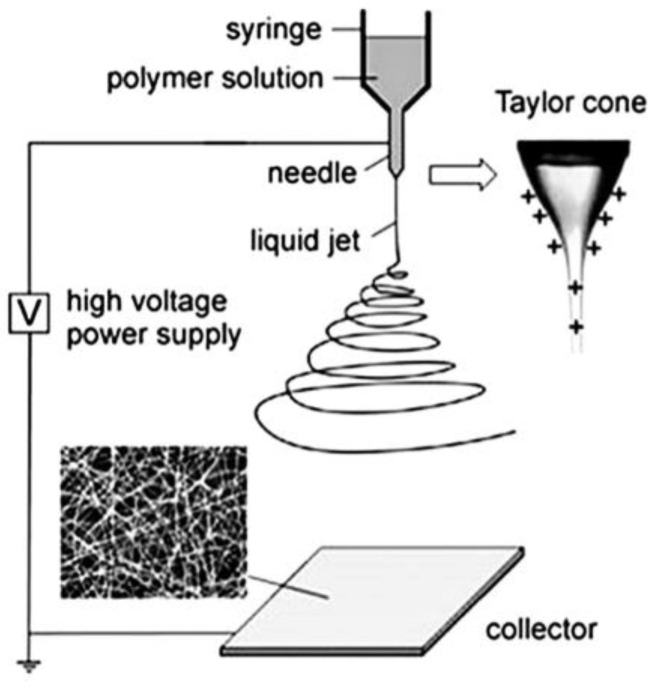
Schematic view of the electrospinning setup for the production of electrospun nanofibers [47].

Owing to the simplicity of the procedure, electrospinning is the most widely discussed method for the production of nano- and microscale polymer fibers. A standard electrospinning setup usually includes a high voltage source, syringe pump with vessel tube, spinneret, and special collector for fiber deposition. A schematic view of the electrospinning setup is shown in Figure 2 [47]. A fundamental concept of the method is based on the stretching of the jet from a polymeric solution under the action of electrostatic forces originating due to the high voltage applied: the charge is prompted inside the polymer creating repulsion forces in a polymeric chain that overcome surface tension and let the charged jet break away. The fibers free from the solvent deposit on the collector. The main types of needle-based and needleless electrospinning techniques together with various types of rotating spinnerets are described in [48].

The use of high voltage values that often reach several tens of kilovolts is one of the key disadvantages of the method, which prevents its use in large-scale production and significantly increases energy consumption costs.

**Figure 3:**
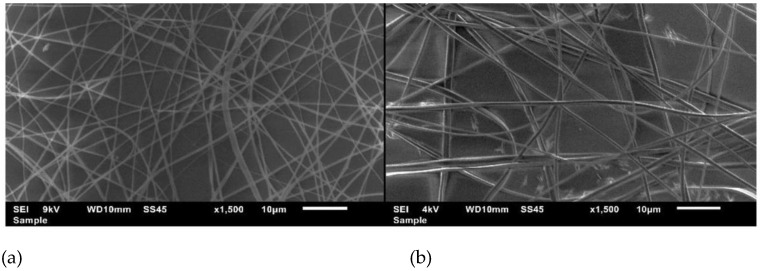
Scanning electron microscopy micrographs of cellulose acetate (CA) nanofibers (**a**) and cellulose acetate/methylene blue (CA/MB) nanofibers (**b**) prepared under the supply voltage, polymer concentration, and flow rate equal to 10 kV, 17%, and 1 mL per h, respectively [49].

Another essential issue of the method is related to the inability to control fiber alignment. Indeed, nanofibrous mats obtained on the electrode-collector consist of randomly arranged filaments with uncontrolled orientation. The images of poorly aligned nanofibers based on cellulose acetate are demonstrated in Figure 3 [49]. Although the thickness of fibers can be controlled in a limited way by varying the properties of the solution (concentration, polymer, and solvent types) and device settings (voltage value), good ordering cannot be achieved in such conditions, which makes the method less advantageous compared to STEP and touch-spinning techniques. In addition, the combination of a large number of factors affecting the stability of the fiber formation, including solvent properties, polymer solution concentration, conductivity, humidity in the chamber, voltage value, etc., greatly complicates the procedure [50,51].

A major limitation of electrospinning application to cellulosic solutions is associated with its insufficient solubility in water and common organic solvents enabled by strong stabilization of the molecules via inter- and intramolecular hydrogen bonds, as well as electrostatic and hydrophobic interactions within the integrated fibrils [52]. Some studies report using solvent systems containing ionic liquids to dissolve cellulose. Thus, N-methylmorpholine-N-oxide (NMMO) [53], tetra(n-butyl) ammonium hydroxide/dimethylsulfoxide (TBAH/DMSO) [54], LiCl/dimethylacetamide (LiCl/DMAc) [55,56], LiOH/urea and NaOH/urea [57], 1-butyl-3-methylimidazolium formate (BMIMFmO) [58] were suggested for the preparation of cellulosic solutions. The altered structure of cellulose derivatives leads to the significant improvement of solubility in common solvent systems. For example, cellulose acetate, one of the most commonly used compounds to obtain electrospun nanofibers, can be dissolved in acetic acid [59], acetone/DMAc [60], or acetone/DMF/water [61] solvents. Hydroxypropyl methylcellulose of high molecular weight was shown to form homogeneous solutions in ethanol in the concentration range of 1–6 wt% [62]. Therefore, the functionalization of cellulose considerably influences the formability of the solutions prepared for electrospinning.

Cellulose, along with other naturally occurring semi-crystalline polysaccharides, such as chitin and its derivative chitosan, is considered a less favorable polymer for direct electrospinning due to its insufficient solubility and the use of non-common solvents, which often involve either highly dielectric or acidic components (acetic and trifluoroacetic acids) affecting further experimental work with biological objects. These technological issues when working with cellulose redirect research interest toward cellulose derivatives as the initial polymers for the production of the solutions for electrospinning. Most studies note that the ultimate pure cellulose-based non-woven materials are likely to be fabricated from cellulose derivatives using a post-treatment after the spinning process. The most commonly applied post-treatment method is the hydrolysis of the fibers in aqueous or alcohol solutions of alkali. Therefore, a lot of research devoted to electrospun cellulose is focusing on the derivatives as the starting components for fiber fabrication with further conversion to cellulose, which is also known as “regenerated cellulose” [63]. In the framework of the current study, special attention will be paid to nanofibers obtained from cellulose derivatives.

## 4. Mechanical Properties of Cellulosic Fibers

The multi-level structure of cellulose-based fibrous materials enables their extraordinary mechanical features, such as high intrinsic stiffness, strength, and modulus, which can be applied to many tissue engineering branches, including bone and cartilage research. Along with the high crystallinity of domains present in specific cellulose kinds and the derivatives, strong hydrogen bonding between cellulose chains plays an important role in the design of high-performance nanomaterials with advanced mechanical features for biomedical applications. Thus, the high values of the elastic modulus (up to 145 GPa) of cellulose I nanocrystals were measured in [64]. Moreover, the additional improvement of the mechanical characteristics is achieved by cellulosic fiber reinforcement with inorganic components. To date, the use of compounds, such as hydroxyapatite (Ca_10_(PO_4_)_6_(OH)_2_) [65], calcium carbonate (CaCO_3_) [66], potassium chloride (KCl) [67], graphene oxide [68], boron nitride [69], bioactive glass [70], etc., have been widely reported in the literature along with the design of complex nanofibrous matrices composed of cellulose (or its derivatives) and various synthetic and natural polymers. Over the past few years, a specific interest has been aroused in composite bacterial cellulose/collagen electrospun materials owing to the substantial elastic modulus up to 115 GPa that bacterial cellulose exhibits and the remarkable interaction between cellulosic nanofibrils with muscle cells similar to that in natural collagen-based ECM [71,72].

Nowadays, one of the key issues of electrospun scaffold design arises from a lack of methods to measure the mechanical properties of single fibers, which is crucial for a basic understanding of the relationship between the properties of a single filament and an ultimate nanofibrous material. Hence, the vast majority of studies provide measurements conducted for electrospun mats rather for single electrospun fibers. Optical techniques, including polarized vibrational spectroscopy and birefringence, are commonly used to give an assessment of polymeric chain orientation necessary for defining the mechanical performance of a single fiber [73]. To demonstrate such a challenging issue, the mechanical properties of cellulose acetate (CA) and cellulose acetate/cellulose nanocrystals (CA/CNCs) fibrous mats can be discussed based on [74]. The authors provide the values of tensile strength and tensile modulus of the materials, which are equal to 12.1 and 1170 MPa respectively for CA-based mats; 16.7 and 1680 MPa for CA/CNCs-based materials, while the measurements of the single fibers remain unperformed. Despite the poor information on the mechanical properties of single cellulosic fibers, nano-tensile testing conducted for a number of polymers is known from the literature. Thus, for instance, collagen/chitosan (1/1)-based fibers of an average diameter of 515 nm showed tensile strength and modulus equal to 60 MPa and 7 MPa, respectively [75]. In [76], thick polycaprolactone fibers with an average diameter of 1400 nm possessed a high tensile modulus of approximately 120 MPa.

**Figure 4:**
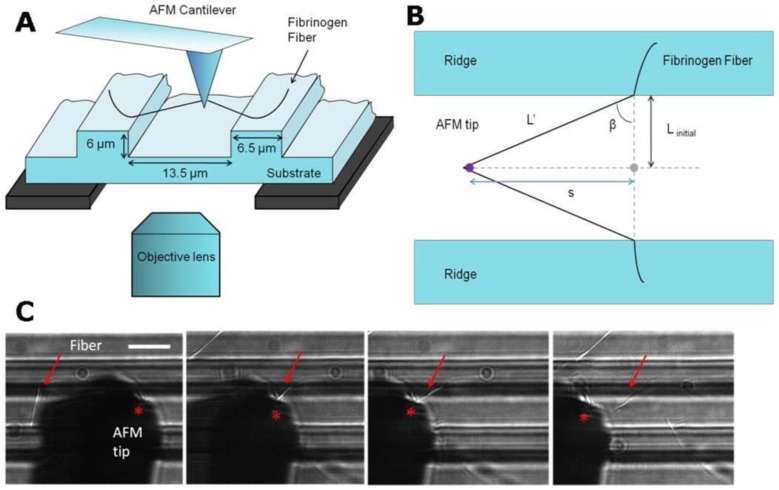
Individual fiber manipulation method: fibrinogen fiber manipulation scheme (**A**); top view scheme (**B**), where L_initial_ and L′ are the lengths of the initial and stretched fiber; optical microscopy visualization of manipulation (**C**) [77].

Along with nano- and micro-tensile testers allowing to investigate electrospun fibers with a diameter <1 µm, wide-angle-X-ray diffraction, atomic force microscopy (AFM) nanoindentation, as well as AFM in combination with optical microscopy are considered efficient techniques for individual fiber study. Baker et al. [77] reported the equipment based on AFM and a fluorescent microscope to perform such measurements. A silicon cantilever of an atomic force microscope was used for lateral deformation of the fibers and detecting the applied forces, while a fluorescent microscope placed under the sample performed the visualization function. A schematic fiber manipulation in accordance with combined atomic force and fluorescent microscopy is presented in Figure 4.

Therefore, it can be easily understood that despite the fascinating mechanical features of electrospun cellulose-based fibers arising from the complex hierarchical organization at nano- and microlevels, the study of individual fiber behavior is a great challenge that hampers a better understanding of the mechanical properties of the polymer materials under study.

## 5. Applicability of Electrospun Nanomaterials in Cell Culturing

Electrospun materials based on cellulose and its derivatives have been widely introduced into clinical practice and investigated as cell carriers for biomedical applications, including multiple areas focusing on tissue regeneration and reconstruction. High demand for cellulosic materials in biomedical engineering led to their extensive investigation in bone and cartilage, skin, liver, pancreatic, skeletal and smooth muscle, vascular tissue engineering, as well as in nervous system research [78]. Such a broad study of cellulosic materials as bioscaffolds involves the assessment of the interaction between various cell cultures and a material that has to provide a suitable environment for cell growth, differentiation, and proliferation due to the combination of unique compositional, structural, and micromechanical features. Today, special attention is awarded to 3D-structured nanofibrous scaffolds with a special diameter-to-porosity ratio, in which the positive influence of the three-dimensional media on processes of cell attachment and growth is realized. In this case, the term “porosity” refers to the distance between individual fibers in fibrous mats that usually should not exceed 80 µm for efficient cell capture and further cell viability. In [79], the authors consider the pore size preferences of several cell lines on different materials in order to adjust the fiber spacing for productive cell culturing. For instance, fibroblasts prefer pores of >90 µm on silicon nitride constructs and, at the same time, the pore size range of 60–150 µm on polymeric matrices, such as poly-L-lactic acid (PLLA). Thus, preferential pore size distributions differ, according to cell line and material used.

**Figure 5:**
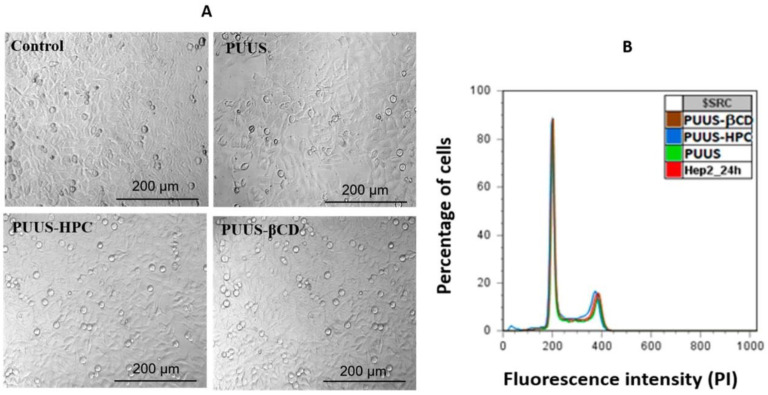
Cell morphology of HEp2 cells on a nanofibrous mat (PUUS) and nanofibrous composite mats (PUUS-HPC and PUUS-βCD) (**A**), cell cycle flow cytometry histograms (**B**) [89].

Among the cell cultures investigated for seeding on nanofibrous scaffolds, fibroblasts [80], embryonic [81], muscle [82], bone marrow-derived [83], endothelial [84], and other cells of mesenchymal origin have been studied. Some examples will be discussed below. It should be taken into account that different cell cultures require different features of the scaffolds and hence scaffold modifications or treatment. Thus, in the work [85] electrospun cellulose-based scaffolds were loaded with bone morphogenetic proteins (rhBMP-2) for further study of bone marrow-derived stem cell osteo-differentiation. The research showed that the combination of fiber alignment and loading with rhBMP-2 induced aligned cortical tissue formation in vivo. Chemically modified through peptide conjugation, cellulose acetate microfibers with a diameter of 100–130 µm were investigated after fetal osteoblasts (hFOB) seeding [86]. Although such microscale fibrous materials do not inherit the dimensions of natural ECM, their considerable effect on cell differentiation is another example of contribution to bone tissue engineering. The integration of human umbilical vein endothelial cells (HUVECs) with cellulose acetate-based dual-polymer electrospun scaffolds treated with fibronectin demonstrated a rapid increase in cell density within 2–4 days and the formation of network-like regions of growth within 10 days [87]. Additionally, the scaffolds with higher mechanical stiffness induced HUVEC growth more productively. In the study [88], the authors provide a detailed investigation of cellulose/conductive polymer (poly(N-vinylpyrrole) and poly(3-hexylthiophene)) nanofibrous mats as functional nerve cell scaffolds. Thus, randomly aligned smooth fibers with a wide thickness distribution of 200–700 nm effectively promoted the proliferation of undifferentiated PC12 cell lines of embryonic origin. The electrospinning technique was used to prepare nanofibrous mats based on hydroxypropyl cellulose (HPC), polyurethane urea siloxane (PUUS), and β-cyclodextrin (βCD) [89]. According to scanning electron microscopy data, randomly aligned nanofibers with a diameter of 110–490 nm were obtained. The bioactivity of the nanomaterials was assessed by the experiments with human dermal fibroblasts (HDF) and human epidermoid cells (HEp2). The results of cytotoxicity tests and cell morphology study are shown in Figure 5.

HPC-containing electrospun mats demonstrate both essential viabilities of the cells determined by the intensity measurements of DNA-stained HEp2 line, and improved mechanical properties resulting in tensile stress values up to 4.8 MPa.

**Figure 6:**
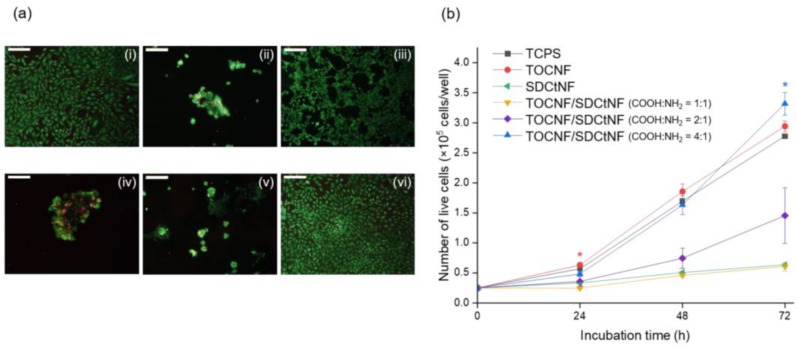
(**a**) Fluorescence images of NIH/3T3 cells cultured for 72 h of (i) TOCNF alone, (ii) SDCtNF alone, (iii) tissue culture plate surface (TCPS), (iv) TOCNF/SDCtNF (COOH:NH_2_ = 1:1), (v) TOCNF/SDCtNF (COOH:NH_2_ = 2:1) and (vi) TOCNF/SDCtNF (COOH:NH_2_ = 4:1) (**b**) Cell counting for each substrate after 24, 48 and 72 h of culture. Mean ± SD, *n* = 3, * *p* < 0.05 vs. TCPS [90].

The efficacy of TEMPO-oxidized cellulose/surface-N-deacetylated chitin (TOCNF/SDCtNF) nanofibrous composites toward NIH/3T3 mouse fibroblasts for skin engineering applications was investigated in [90]. The morphology of fibroblasts on single TOCNF and SDCtNF substrates, as well as on polymer blend, was observed using optical microscopy with subsequent visualization by live/dead staining with calcein AM (live) and ethidium homodimer III (dead) after 72 h of incubation (Figure 6). The authors reported the increased cell growth for the composite TOCNF/SDCtNF substrates determined by the presence of COOH/NH_2_ (4/1) groups responsible for surface charge regulation and hence the improved protein adsorption to direct integrin binding.

The research conducted by the authors in [90] reflects the importance of studying the cell adhesion mechanism starting with the simple electrostatic interaction between the cells and culturing surfaces carrying a slight charge ensured by the presence of specific functional groups. A number of papers devoted to cell adhesion study provide data on the relationship between the ζ-potential of surfaces and the types of cells to be seeded. Chang et al. [91] demonstrated that the tunable ratio of NH_2_ and COOH groups in self-assembled monolayers had an effect on the surface potential of epithelial cell density ensured by the higher adsorption kinetics of laminin on the surface. In contrast, the adsorption of the negatively charged fibronectin made the ECM less homogeneous for fibroblast adhesion. The effect of negative charge incorporation on protein adsorption was also observed in [92]. 

The study [93] demonstrates the potential feasibility of 2D and 3D cellulose acetate/pullulan (CA/PULL) constructs fabricated via conventional and wet electrospinning set-ups in skin tissue research. Relatively thick fibers with a diameter of 0.11–33.17 µm and fiber spacing of up to 200 µm were obtained by varying CA and PULL content, and the samples with the 50/50-ratio were used to seed mouse fibroblastic cell line (L929). Cell morphology on CA/PULL-based electrospun scaffolds after PULL removal was observed by scanning electron microscopy (SEM) at different incubation periods (1, 4, 7, and 14 days), which revealed the cell attachment and expansion after 1- and 4- day incubation. The migration of the cells examined by confocal laser scanning microscopy with Z-stacking analysis (10–190 µm depth) proved the migration inside the material on the 7th day.

The viability and expansion of HUVECs on cellulose acetate and core-shell cellulose acetate/polycaprolactone (CA/PCL) fibers were analyzed by Khalf et al. [94]. SEM study for both hollow and core-shell structures prepared by coaxial electrospinning method revealed sufficient cell attachment and entanglement throughout the matrix with no difference in cell morphology and expansion compared to tissue culture plastic surface. Cellulose acetate fibers with loaded PCL cores were shown to maintain better elastic elongation compared with hollow cellulose acetate electrospun nanofibers.

Carboxymethyl cellulose-containing nanofibers with advanced antimicrobial properties were prepared by electrospinning from carboxymethyl cellulose/polyvinyl alcohol (20/80) solutions with the loading of antimicrobial agent colistin and citric acid-based quantum dots as crosslinked agents [95]. The cytotoxicity of the fibers was assessed with human foreskin fibroblast (HFF-1) culturing and an MTT test. Thus, the results showed the cell viability remained over 80% after 5 days. The suitable cell proliferation observed by microscopy study together with the antimicrobial effect of colistin enables scaffold application in skin tissue engineering as wound dressings.

Overall, a broad spectrum of cell cultures is presented in the literature, depending on the target direction of biomedical research. The ways of scaffold modification with bioactive organic molecules (proteins and peptides) or polymer combinations for cell adhesion improvement are remaining crucial. At the same time, the adjustment of pore size distribution required for specific cell lines needs to be implemented.

## 6. Conclusions

The studies of the last 20 years demonstrate that electrospun nanomaterials based on cellulose and its derivatives attract huge research interest for multiple clinical applications, including such advanced areas as cell culturing for tissue engineering. The unique mechanical stability of cellulosic filaments that arises from the hierarchical structure of polymers and the presence of a variable fraction of crystalline domains, as well as the biocompatibility with various cell lines led to the development of the technology for the production of nanofibrous ECM-like polymeric matrices. Electrospinning techniques frequently discussed in the literature provide opportunities to fabricate non-woven scaffolds with submicron thickness and sufficient pore interconnectivity based on cellulose derivatives. The difficulties associated with the implementation of electrospinning from pure cellulose solutions require the use of atypical solvents, for example, ionic liquids or acids, which redirects the attention of researchers toward cellulose derivatives (cellulose acetate, carboxymethylcellulose, hydroxypropyl cellulose, etc.) that can be easily converted to cellulose by hydrolysis if needed. A lack of fiber alignment and pore size control in electrospun mats, along with very limited abilities to observe individual fiber micromechanics remain unresolved issues of electrospun scaffold manufacture. Despite these technological aspects, the application of electrospun scaffolds has already shown fairly successful results at the initial stages of tissue engineering research and hence defined a great potential for further study and improvement. Thus, 2D and 3D nanofiber matrices based on cellulose, cellulose derivatives, or their composites with other polymers exhibit suitable environment for the attachment, growth, and proliferation of endothelial, muscle cells, osteoblasts, fibroblasts, etc., which allows for scaffold applications in soft and hard tissue research.

## Figures and Tables

**Table 1:** Cellulose derivatives widely discussed in the literature *.

Derivative Groups	Substance	Functional Groups
Carboxyalkyl	Carboxymethylcellulose	CH_2_COONa
Alkyl	Methylcellulose	CH_3_
	Ethylcellulose	C_2_H_5_
	Methyl ethylcellulose	CH_3_/C_2_H_5_
Hydroxyalkyl	Hydroxyethylcellulose	C_2_H_4_OH
	Hydroxyethyl methylcellulose	C_2_H_4_OH/CH_3_
	Hydroxypropyl cellulose	CH_2_CH(OH)CH_3_
	Hydroxypropyl methylcellulose	CH_2_CH(OH)CH_3_/CH_3_
	Ethyl hydroxyethylcellulose	C_2_H_5_/C_2_H_4_OH
Organic substituents	Cellulose acetate	CH_3_CO
	Cellulose propionate	C_2_H_5_CO
	Cellulose xanthate	OCS_2_Na
Inorganic substituents	Cellulose phosphate	H_2_PO_3_
	Cellulose sulfate	SO_3_H
	Cellulose nitrate	NO_2_

* Adapted with permission from Ref. [31]. Copyright 2023, Wiley-VCH Verlag.

## Data Availability

All the data is available within the manuscript.
